# Relationship between Chalkiness and the Structural and Physicochemical Properties of Rice Starch at Different Nighttime Temperatures during the Early Grain-Filling Stage

**DOI:** 10.3390/foods13101516

**Published:** 2024-05-13

**Authors:** Changzhi Long, Yanli Du, Mingyang Zeng, Xueyun Deng, Zhengwei Zhang, Dong Liu, Yongjun Zeng

**Affiliations:** 1 Key Laboratory of Crop Physiology, Ecology and Genetic Breeding, Ministry of Education, Jiangxi Agricultural University, Nanchang 330045, China; longchangzhy@163.com (C.L.); dyli1992@163.com (Y.D.); myzengbio@163.com (M.Z.); dxy2020jx@163.com (X.D.); 15551551951@163.com (Z.Z.); liudjxau@126.com (D.L.); 2 Lushan Botanical Garden, Chinese Academy of Sciences, Lushan 332900, China

**Keywords:** chalkiness, starch fine structure, different nighttime temperatures, early grain-filling stage

## Abstract

The chalkiness, starch fine structure, and physiochemical properties of rice starch were analyzed and their correlations were investigated under different nighttime temperatures during the early grain-filling stage. Compared to MT, medium temperature (MT) and low (LNT) and high (HNT) nighttime temperatures resulted in an increased chalky grain rate (CGR) and chalkiness degree (CD). LNT mainly affected the chalkiness by increasing peak1 (short branch chains of amylopectin), the branching degree, and the proportion of small starch granules but decreasing peak2 (long branch chains of amylopectin) and peak3 (amylose branches). This altered the pasting properties, such as by increasing the peak viscosity and final viscosity. However, HNT mainly affected the chalkiness by increasing peak2 and the crystallinity degree but decreasing peak1 and peak3. Regarding the thermal properties, HNT also elevated peak and conclusion temperatures. The CGR and CD were significantly and positively correlated with the proportions of small and medium starch granules, peak1, branching degree, gelatinization enthalpy, setback viscosity, and pasting time but markedly and negatively correlated with the proportion of large starch granules, amylose content, peak3, peak viscosity, and breakdown viscosity. These findings suggest that LNT and HNT disrupted the starch structure, resulting in increased chalkiness. However, their mechanisms of action differ.

## 1. Introduction

Rice is one of the world’s most economically crucial food crops and plays a vital role in ensuring food security for the future. The demand for high-quality rice has increased recently, with an improvement in people’s living standards [1]. The goal of rice production has shifted from yield first to yield and quality in parallel. Rice quality consists of its appearance, processing, nutrition, and eating and cooking qualities. Appearance is amongst the most critical determinants of rice quality and is closely related to the processing quality and eating and cooking quality [2].

The appearance of rice is mainly determined by its chalkiness. The chalky, opaque portion of the rice grain deteriorates the appearance and eating and cooking quality [3]. Excessive chalkiness of rice will lead to poor processing quality in rice, which is due to the high chalkiness of the internal arrangement of the grain being loose, and the texture of the rice is loose [4]. It is easy to break during the milling process, which reduces the processing quality of rice. High or low temperatures have repeatedly been demonstrated to adversely affect rice quality, especially the CGR and CD, due to changes in the biosynthesis rates of starch and other storage compounds during the grain-filling stage (GFS) [5]. The temperature mainly affects the rice chalkiness through a certain period during the GFS. Wakamatsu et al. [6] reported that temperature changes most significantly affected rice quality within 20 days after heading. Siddik et al. [7] also found that rice quality was most sensitive to high or low temperatures during a 7–14 day period after heading. Moreover, high temperatures more severely affected chalkiness than low temperatures [7]. Under high or low temperatures, the starch granules in rice endosperm were inconsistent in shape and loosely arranged, resulting in more gaps among the starch granules, thereby reflecting more light and thus resulting in chalkiness [8].

Starch, which is a major storage carbohydrate in rice, consists of amylose and amylopectin, accounting for 80–90% of the total weight. The fine structure and physicochemical properties of starch are the major determinants that affect the rice quality [9]. The starch fine structure is closely related to the development and occurrence of rice chalkiness [10]. An increase in the number of short chains in amylopectin, crystallinity, and the order of crystallinity, but a decrease in the number of long chains in amylopectin and the ratio of amorphous to ordered starch structure, leads to a deterioration in rice quality by enhancing its chalkiness [11]. In addition, the size distribution of the starch granules is also one of the critical factors affecting rice chalkiness [12]. In a study, scanning electronic microscopy (SEM) observation showed a deficient uniformity and regularity of starch granules, resulting in the altered transmission of light through the endosperm, thereby increasing the CGR and CD [13]. However, the fine structure and physicochemical properties of starch, which are some of the most critical determinants of chalkiness, are influenced by environmental conditions and are genetically regulated [8,9].

Changes in temperature greatly affect the yield and quality of rice. The ambient temperature during the GFS in rice is one of the most critical environmental factors affecting the fine structure of the starch in the endosperm [14]. High temperatures inhibit the activities of granule-bound starch synthase (GBSS) and starch branching enzymes (SBEs). Therefore, the ratio of short to long chains of amylopectin is increased, but the AC is decreased, thereby increasing the CGR and CD [8,15]. Additionally, high temperatures stimulate the expression of amylase-encoding genes and increased amylase activity, leading to starch degradation and the formation of starch granules of inconsistent size [16,17]. Low temperatures increase the length of amylopectin short chains while decreasing the length of amylopectin long chains and relative crystallinity of the starch, thus altering its crystal structure [18]. These modifications in the starch structure reduce the uniformity and regularity of starch granules and widen the gaps among them [19].

Elevated ambient temperatures during GFS affect the fine structure of starch and increase the CGR and CD [9]. Compared with the daily maximum temperature and daily average temperature, nighttime temperature has a greater effect on the formation of chalkiness in rice [20]. Cheng et al. [21] showed that the effect of nighttime temperature on rice chalkiness was more pronounced after a 7-day period after heading. At present, the influence of variations in temperature on the chalkiness and the physicochemical properties of starch has mainly been studied under extended treatment conditions throughout the GFS. However, the formation of rice chalkiness and its relationship with the starch fine structure and physicochemical properties under short-term low nighttime temperature (LNT) or high nighttime temperature (HNT) treatments are still unclear. Therefore, LNT and HNT treatments were performed between the 7th and 14th day after heading to evaluate them. Our results provide useful information for understanding the underlying causes of rice chalkiness occurrence under varying nocturnal temperatures at the early GFS.

## 2. Materials and Methods

### 2.1. Plant Materials and Experimental Design

The experiments were conducted at the Science and Technology Park of Jiangxi Agricultural University (115°49’53″ E, 28°46’8″ N), Nanchang, Jiangxi Province, China. Two indica rice cultivars, Jiuxiangzhan (JXZ) and Huanghuazhan (HHZ), were used. The soil was pre-experimentally air-dried and filtered through a 5 mm sieve, and 8 kg of it was added to each pot. The chemical properties of the soil were 30.12 g·kg^−1^ organic matter, 2.27 g·kg^−1^ total N, 97.71 mg·kg^−1^ alkaline hydrolyzable N, 100.73 mg·kg^−1^ available K, and 27.37 mg·kg^−1^ available P.

The pot-planting method was used to cultivate the rice plants, as described previously [22]. Briefly, the seedlings were transplanted to the pots (length, 27.5 cm; width, 21.0 cm; height, 32.0 cm) 28 days after sowing. The seedlings were planted in 2 holes per pot, with 2 seedlings per hole, and 40 pots were used for each treatment, which was performed in triplicate. The N was applied at a rate of 2.61 g N pot^−1^, and the basal, tillering and panicle fertilizer were applied in 5:2:3 ratio. The application of N, P (as P_2_O_5_), and K (as K_2_O) was in the ratio of 2:1:2. P was applied only as a basal fertilizer, but K was applied as 70% basal and 30% as panicle fertilizer. Other management techniques, such as pest and disease control, were implemented based on the high-yield and -quality cultivation program.

On the 7th day after heading, the rice plants were moved to artificially controlled-climate chambers for temperature treatment, with each chamber divided into two halves for the two cultivars, JXZ and HHZ. Temperature settings during the grain-filling stages were set in reference to the manner of Du et al. [23]. The rice plants were treated with different nighttime temperatures (18:00–06:00 h) for 7 days as follows: (i) medium temperature (MT = 30 °C–25 °C); (ii) low nighttime temperature (LNT = 25 °C–20 °C); and (iii) high nighttime temperature (HNT = 35 °C–30 °C). The daytime (06:00–18:00 h) temperatures were 27 °C–32 °C on all days. The divergent temperatures varied at intervals of 1 °C linearly during the temperature treatment period. The temperature settings are detailed in Table A1.

### 2.2. Determination of CGR and CD

The determination of chalkiness was performed using the Wanshen Rice Analysis System. Refined, whole rice grains from the triplicates of each treatment were analyzed, and their average values were obtained to determine the CGR and CD as follows:

CGR (%) = Number of chalky grains/Number of observed grains × 100%(1)

CD (%) = Chalky size × CGR(2)

### 2.3. Determination of AC

The AC was determined based on the methods described by the National Standard of Rice Quality Evaluation “GB/T17871-2017” [24], the People’s Republic of China.

### 2.4. Ultrastructural Observations of Starch Granules

The ultrastructural properties of the amyloplasts in the endosperm were determined based on a previously described method [11]. The arrangement and surface structure of starch granules were observed on a GeminiSEM 300 instrument (Zeiss, Oberkochen, Germany).

### 2.5. Determination of Granule Size

The granule size was determined using the Mastersizer 3000 (Malvern Panalytical Ltd., Worcestershire, UK) according to a previously described method [25]. Based on the volume, surface area, and proportion, starch granules were categorized into small and medium (<10 μm) or large (>10 μm).

### 2.6. Analysis of the X-ray Diffraction (XRD) Patterns and Determination of the Branching Degree

The XRD of the starch molecules was performed with an X’Pert Pro X-ray diffractometer (PANalytical, EA Almelo, The Netherlands) under the following conditions: power at 1600 W (40 kW × 40 mA) using a scanning range of 4°–60°, step size of 0.02°, and scan speed of 4°/min. The parameters of the degree of crystallinity (%), crystalline morphology, and diffraction peaks at 2θ value (angle) were calculated using the software MDI Jade 6 (Materials Data, Inc, Livermore, CA, USA) Jade 6.0. The starch branching distribution was determined via 1H NMR analysis (Bruker BioSpin GmbH, Ettlingen, Germany) with a scan number of 32, a resonance RF of 500.23 MHz, and an NMR spectrum of 1H. The data were analyzed using the MestReNova 14.0 software.

Branching degree (%) = A/(A+B) × 100%(3)

A and B indicate the peak areas of the α-1,6 and α-1,4 linkages, respectively.

### 2.7. Molecular Weight Distribution of Starch

The starch dissolved in a DMSO-LiBr solution was debranched using isoamylase according to previous methods [26], and the components’ molecular weights were ascertained using a U3000 GPC (Thermo Fisher Scientific, Waltham, MA, USA) and an OPTILAB^®^ T-rEX™ differential detector (Wyatt Technology, Goleta, CA, USA). The chromatographic data were processed using the ASTRA 6.1 software. Pullulan was used as a molecular weight standard consisting of 342, 3650, 21,000, 131,400, 610,500, 821,700, and 3,755,000 Da.

### 2.8. Measurement of Thermal Properties

The DSC 200 F3 differential calorimetric scanner (NETZSCH, Waldkraiburg, Germany) was employed to ascertain the changes in the enthalpy of the starch by scanning the heat at a temperature-change rate of 10 °C/min from 30 °C–105 °C. The heat change data were then analyzed using Universal Analysis/Proteus Thermal Analysis 2000 software. The parameters of onset temperature (To), peak temperature (Tp), conclusion temperature (Tc), and enthalpy of gelatinization (ΔH) during the process of the phase change in the sample were calculated separately [25].

### 2.9. Determination of Pasting Properties

The pasting properties of starch were determined using a Starch Master TM 17,133 Rapid Viscosity Analyzer (Newport Scientific Pvt. Ltd., Warier Wood, Australia). The RVA spectral characteristics included peak viscosity (PV), trough viscosity (TV), final viscosity (FV), breakdown viscosity (BD), setback viscosity (SB), pasting temperature (PaT), and pasting time (PT).

### 2.10. Statistical Analysis

All data for each treatment are represented as the means of three replicates. Statistical analyses were performed using SPSS 22.0 software (SPSS Inc., Chicago, IL, USA) using the least significant differences (LSD) method at the *p* < 0.05 level. Pearson correlation analysis (PCA) was performed to assess the correlation between chalkiness and the fine structure and the physicochemical properties of rice starch. The differences at *p* levels < 0.05 or <0.01 were considered statistically significant.

## 3. Results and Discussion

### 3.1. Chalky Rice Grain Rate (CGR) and Chalkiness Degree (CD)

**Figure 1:**
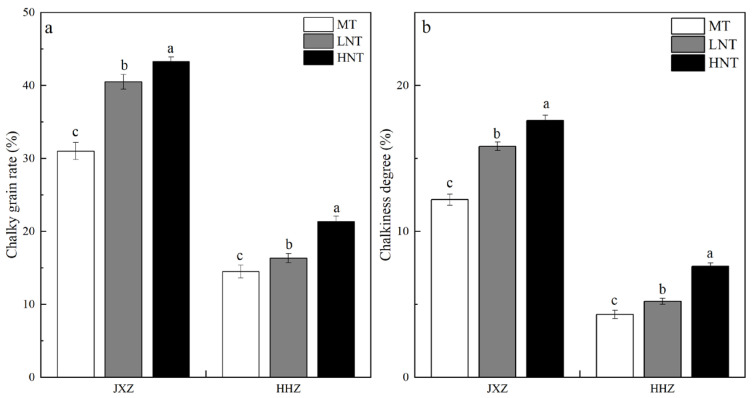
Effects of different nighttime temperatures on chalky grain rate (**a**) and chalkiness degree (**b**). Abbreviations: JXZ, Jiuxiangzhan; HHZ, Huanghuazhan; MT, medium temperature; LNT, low nighttime temperature; HNT, high nighttime temperature. Different lowercase letters indicate a significance at *p* < 0.05 for the same cultivars in the same group under varying nighttime temperatures.

The CGR and CD of milled rice observed at different nighttime temperatures during the early GFS are presented in Figure 1a,b. Both the LNT and HNT significantly increased the CGR and CD of the two rice cultivars (JXZ and HHZ), and the effect of the HNT was greater than that of the LNT. This was in agreement with the study conducted by Meng et al. [27]. Under LNTs, the impediment of the growth and development of grains leads to the development of chalkiness in the starch [28]. Under HNTs, the grain filling rate is accelerated, which leads to the disruption of starch arrangement in the grains, resulting in the formation of chalkiness [29].

### 3.2. Amylose Content (AC)

The different nighttime temperatures significantly altered the AC during the early GFS (Table 1). Under the LNT, the change in the AC in the JXZ variety was insignificant but was decreased markedly by 6.24% in the HHZ variety. The activity of GBSS, an enzyme critical for amylose biosynthesis, determines the AC [30]. Significant variabilities in the activities of the GBSS in different rice cultivars were observed under low temperatures [31,32]. Thus, the low activity of GBSS in the HHZ variety may have resulted in a remarkable decrease in the AC, which may be among the main determinants of the AC in the JXZ and HHZ varieties. On the contrary, under the HNT, the AC decreased in both rice cultivars, which could have been due to the reduced activity of GBSS under high temperatures, as reported earlier [33].

### 3.3. Morphology and Arrangement of Starch Granules

**Figure 2:**
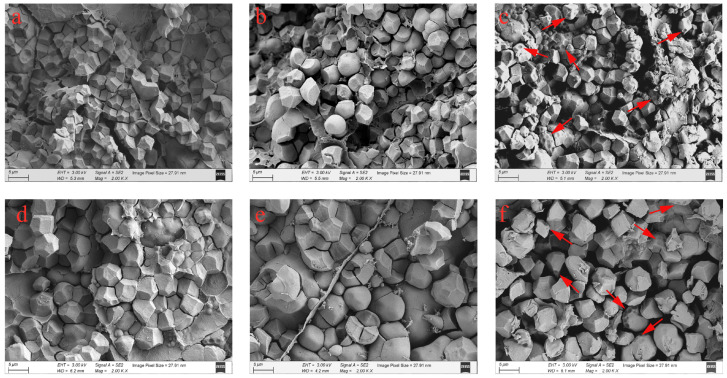
Scanning electron microscope photographs of rice grains from plants exposed to MT, LNT, and HNT: the grains of JXZ (**a**) and HHZ (**d**) varieties under MT; JXZ (**b**) and HHZ (**e**) varieties under LNT; and JXZ (**c**) and HHZ (**f**) varieties under HNT. The arrows indicate the small pits on the surfaces of the starch granules. Abbreviations: JXZ, Jiuxiangzhan; HHZ, Huanghuazhan. MT, medium temperature; LNT, low nighttime temperature; HNT, high nighttime temperature.

The morphology and arrangement of starch granules in the endosperm determine the rice chalkiness [34]. In this study, the morphological characteristics and arrangement of the starch granules were observed through SEM. As shown in Figure 2, under the MT, the amyloplasts in the endosperms of JXZ and HHZ were complex, with several similarly sized, polyhedral starch granules that were arranged closely. In contrast, the starch granules in the grains of plants exposed to the LNT and HNT were poorly developed, with spherical shapes, heterogeneous sizes, and large airspaces among them, thereby reducing the uniformity and regularity in their arrangement. Additionally, numerous small pits could be observed on the surfaces of the starch granules under the HNT. The starch granules have been reported to be loosely arranged, along with an increase in the number of the small pits on their smooth surface under low or high temperatures [17,18]. However, the underlying mechanisms vary under the different temperature regimes. Under the LNT, the described phenomenon was caused by a delay in amyloplast development due to the reduced translocation of water-soluble carbohydrates [35]. However, the HNT induced the expression of the amylase-encoding genes, which enhanced the α-amylase activity in the endosperm, resulting in the appearance of the numerous small pits on the surfaces of the starch granules [17].

### 3.4. Size-Based Composition of Starch Granules

A similar trend was observed in the composition of the starch granules based on their sizes in the two indica cultivars (Table 1). The LNT significantly increased the proportion of small and medium-sized (<10 μm) starch granules in the JXZ and HHZ varieties compared to the MT, but that of large (>10 μm) starch granules was reduced markedly. The proportions of the numbers and surface areas of the starch granules also exhibited an identical trend, with those of the large starch granules reduced by 14.05% and 24.14% in the JXZ variety, respectively, and by 20.27% and 26.25% in the HHZ variety, respectively. Similar results were observed under low temperatures at the GFS, which inhibited the enzymes related to the biosynthesis and accumulation of starch, resulting in an elevation in the number of small and medium-sized starch granules [36,37]. Meantime, the LNT delayed the development of starch granules in the endosperm and inhibited the absorption and accumulation of assimilates, thereby blocking the biosynthesis of large starch granules [19,35]. The HNT markedly decreased the proportion of the surface area of large starch granules in the JXZ variety while its effect was not prominent in the HHZ variety. The activity of the amylase was elevated under high temperatures during the GFS, which accelerated the enzymatic hydrolysis of starch granules [17], resulting in pitting on their surfaces and thereby widening the gaps among the starch granules and increasing the grain chalkiness. Therefore, an enhancement in the proportion of small and medium starch granules when compared to large starch granules contributed to a higher chalkiness under the LNT and HNT.

### 3.5. Molecular Structure of Starch

**Figure 3:**
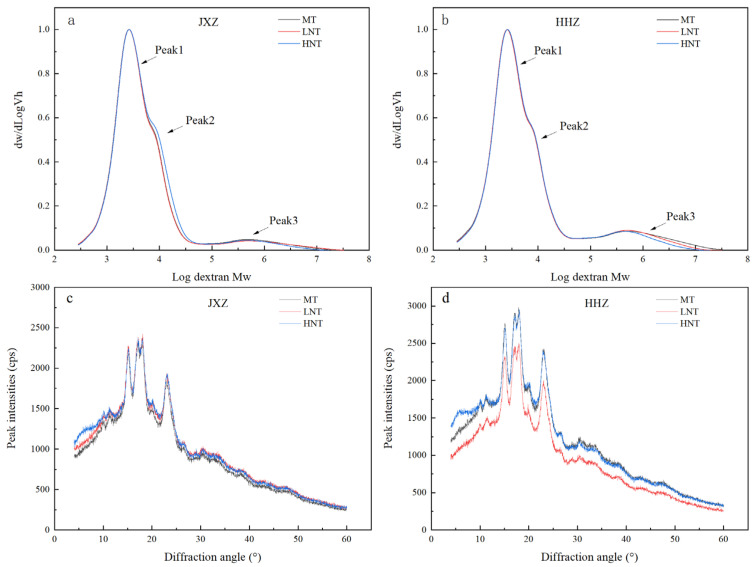
Effects of different nighttime temperatures on GPC profiles (**a**,**b**) and XRD patterns (**c**,**d**). Abbreviations: JXZ, Jiuxiangzhan; HHZ, Huanghuazhan. MT, medium temperature; LNT, low nighttime temperature; HNT, high nighttime temperature.

A trimodal distribution consisting of peak1, peak2, and peak3, corresponding to the low, middle, and high molecular weights, was observed through gel permeation chromatography (GPC) analysis (Figure 3a,b). Peak1 and peak2 corresponded to the short (A and short B chains) and long (long B chain) branch chains of amylopectin, respectively, and peak3 corresponded to the amylose branches [38]. The GPC-related parameters of starch under the LNT and HNT are shown in Table 2. Under the LNT, peak1 increased markedly while peak2 and peak3 decreased significantly in the JXZ variety; in contrast, no changes were observed in the HHZ variety. Low temperatures enhanced the activities of starch branching enzymes [39], which was in agreement with the remarkable decrease in peak2 and peak3 under the LNT observed in our study. On the contrary, peak1 and peak3 decreased in the JXZ variety under the HNT while the opposite was observed for peak2. Similarly, peak2 increased, but peak3 decreased in the HHZ variety. This suggests that the HNT reduced the proportion of short branch chains of amylopectin and amylose branches. Possibly, the HNT rapidly inhibited the activities of soluble starch synthase (SSS) and the starch branching enzymes (SBEs), thus decreasing peak1 and increasing peak2 [39,40].

### 3.6. Crystallinity and Branching Degree

The XRD showed a typical A-type diffraction pattern under the LNT and HNT during the early GFS (Figure 3c,d), indicating that they did not alter the crystalline structure as suggested by previous studies [18,19]. The LNT and HNT treatments did not alter the crystalline structure but did alter the degree of crystallinity. The crystallinity of the starch was mainly determined based on the chain length, crystal order, AC of the amylopectin double helix, and the H-bond or glycosidic bond interactions between them [41]. In our study, the LNT did not markedly affect the crystallinity degree (Table 2). However, under the HNT treatment, the crystallinity degree was markedly increased by 17.72% and 24.24% in the JXZ and HHZ varieties, respectively. Similar results were also observed by Yang et al. [13]. The formation of microcrystalline structures was promoted by the alignment of starch molecules as the number of short chains of amylopectin (peak1, Table 2) and the amylose branches (peak3, Table 2) decreased [42,43], which may have been the reason behind the higher CGR and CD observed with the HNT compared to the LNT.

The LNT significantly elevated the branching degree of the starch in the JXZ variety (Table 2), which may have been due to the high activity of the starch branching enzymes under the LNT, resulting in the breakage of the α-(1,4)-glycosidic linkages to form α-(1,6)-glycosidic bonds, thereby increasing the branching degree. On the contrary, the HNT markedly decreased the branching degree of starch in the JXZ variety but had the opposite effect in the HHZ variety. This may have been due to different heat tolerance capabilities between the two cultivars, with the HHZ variety being more resistant. As a result, the activities of starch branching enzymes in the JXZ variety were inhibited under the HNT, but those in the HHZ variety remained highly active. In addition, these differences may also have been related to the insufficient supply of precursors, such as sucrose, in the JXZ variety, for starch biosynthesis, leading to a disorganization of the starch molecules.

### 3.7. Pasting Properties of Starch

The paste viscosity of rice starch varied markedly under different nighttime temperatures at the early GFS (Table 3). Compared to the MT, the LNT significantly increased the PV, TV, FV, and SB in the JXZ variety while the PaT was remarkably decreased. Additionally, the PV and FV were significantly increased in the HHZ variety while no differences were observed in the other parameters. Since the RVA profile characteristics were previously proven to be closely correlated with the cold tolerance of rice cultivars [44], it is speculated that the JXZ variety may be more sensitive to LNT than the HHZ variety. However, the variations in the starch pasting properties between the two cultivars were inconsistent under the HNT, with the PV, TV, FV, SB, and Pt increasing and the PaT decreasing markedly in the JXZ variety and the opposite being observed in the HHZ variety. The starch pasting properties were tightly associated with the AC [45]. The variations in the AC between the JXZ and HHZ varieties may have led to the differences in the starch pasting properties in the two cultivars in response to the HNT.

### 3.8. Thermal Properties of Starch

The thermal properties of starch reflect the transformation of crystal structures during starch pasting in rice [41]. The T_o_, T_p_, T_c_, and ΔH values of rice starch obtained in this study are summarized in Table 4. No significant change in the ΔH was detected under the LNT compared to the MT. However, the T_o_ was reduced by 1.0 and 1.63 °C in the JXZ and HHZ varieties under LNT, respectively, while T_c_ was decreased by 1.45 °C in the JXZ variety. Similarly, under the HNT, there was no significant change in the ΔH. However, the T_p_ in the JXZ and HHZ varieties increased markedly by 0.21 and 1.43 °C, respectively. Moreover, the T_c_ was enhanced by 1.53 °C in the HHZ variety. This was potentially a result of the lower AC, higher relative crystallinity, and lower peak3 under the HNT (Table 1 and Table 2), as observed in previous studies [45]. The LNT reduced the PaT, indicating that the double helix of starch unfolded and that the minimum temperature needed for the disruption of the crystal structure was reduced. However, the opposite trend was observed under the HNT. This indicates that the LNT and HNT altered the structure and thermal properties of the starch, but with apparent differences in the underlying mechanisms.

### 3.9. Correlation between Chalkiness and the Structural, Thermal, and Pasting Properties of Starch

Chalkiness is mainly regulated by the arrangement and fine structure of starch granules, which affects the physicochemical properties [11,46]. In this study, chalkiness was significantly correlated with the starch fine structure and its thermal and pasting properties (Table 5). The CGR and CD were negatively correlated with the AC but positively with the branching degree and the ΔH. The PV and BD were negatively correlated, but the SB and Pt were positively correlated with the CGR and CD. The nighttime temperature-induced stress increased the rice chalkiness by mainly altering the fine structure of the starch and also altering its physicochemical properties. Previous studies have found that starch structure is closely related to physicochemical properties, with lower amylose content leading to lower internal stability of starch granules whereas a lower percentage of short branch chains of amylopectin is associated with higher ΔH and PV [47]. Patindol et al. [14]. showed that starch fine structure at HNT during the night, with a decrease in the percentage of short branch chains of amylopectin and an increase in the percentage of long branch chains of amylopectin, led to an increase in crystallinity and PV and DB. Our results indicate that the CGR and CD were positively correlated with the proportions of volume (V) < 10 μm, surface area (S) < 10 μm, and numbers (N) < 10 μm while the opposite, i.e., V >10 μm, S >10 μm, and N >10 μm, was observed under either the LNT or HNT. Cao et al. [12] demonstrated that an increase in starch grain size helped to reduce the chalkiness of rice. HNT under the grain-filling stage led to a smaller diameter of the starch granules, which may have been related to increased α-amylase activity [48] and the inhibition of starch synthase [49]. LNT delays the development of amyloplasts and reduces the particle sizes of starch granules [28]. At present, LNT also inhibits nutrient translocation into the endosperm [35], leading to insufficient starch perfusion and uneven starch granule size. In addition, the increase in the proportions of small- and medium-sized starch granules indicates that the granules were filled with less starch and were less homogeneous, leading to more prominent gaps among them. In summary, the LNT or HNT caused abnormal starch biosynthesis and changed the chain length distributions and branching degree of amylopectin, thus resulting in an altered starch fine structure. All of these disrupted the organized nature of the starch granules and contributed to the increase in the CGR and CD.

## 4. Conclusions

The assessment of the effects of LNT and HNT during the early GFS on the fine structure and physicochemical properties of starch is essential for understanding the mechanisms underlying the occurrence of chalkiness in rice. Our results indicate that the LNT and HNT in the study significantly increased chalkiness but demonstrated distinct mechanisms of action. The LNT increased peak1 and the branching degree but lowered peak2 and peak3, thereby increasing the proportion of small- and medium-sized starch granules. On the other hand, the HNT decreased peak1 and peak3 but increased peak2 and the crystallinity degree, resulting in an increase in the percentage of the surface area of small- and medium-sized starch granules. These factors altered the fine structure of the starch, thereby reducing the uniformity and arrangement of the starch granules, which eventually led to an increase in the CGR and CD under different nighttime temperatures. These findings could help further investigations into the mechanisms by which varied nighttime temperatures at the early GFS increase the occurrence of chalkiness by affecting the physicochemical properties of starch in rice.

## Figures and Tables

**Table 1:** Effects of different nighttime temperatures on amylose content and starch granule size distribution.

Cultivars	Treatments	Amylose Content (%)	Volume Percentage (%)		Surface Area Percentage (%)		Number Percentage (%)	
			<10 μm	>10 μm	<10 μm	>10 μm	<10 μm	>10 μm
JXZ	MT	17.11 a	66.10 b	33.90 a	90.75 b	9.25 a	99.71 b	0.29 a
	LNT	16.62 a	67.47 a	32.53 b	92.05 a	7.95 b	99.78 a	0.22 b
	HNT	15.69 b	67.02 ab	32.98 ab	91.92 a	8.08 b	99.75 ab	0.25 ab
HHZ	MT	21.16 a	56.86 b	43.14 a	84.12 b	15.88 a	99.20 b	0.80 a
	LNT	19.84 b	63.48 a	36.52 b	87.34 a	12.66 b	99.41 a	0.59 b
	HNT	19.04 b	58.48 ab	41.52 ab	85.29 ab	14.71 ab	99.17 b	0.83 a

Different lowercase letters indicate a significance at *p* < 0.05 for the same cultivars in the same column under varying management. Abbreviations: JXZ, Jiuxiangzhan; HHZ, Huanghuazhan. MT, medium temperature; LNT, low nighttime temperature; HNT, high nighttime temperature.

**Table 2:** Effects of different nighttime temperatures on GPC parameters, crystallinity degree, and branching degree of rice starch.

Cultivars	Treatments	GPC Peak Area (%)			Crystallinity Degree (%)	Branching Degree (%)
		Peak1	Peak2	Peak3		
JXZ	MT	61.64 b	22.41 b	15.95 a	27.25 b	3.82 b
	LNT	62.48 a	22.05 c	15.47 b	26.31 b	3.98 a
	HNT	60.09 c	25.15 a	14.76 c	32.08 a	3.59 c
HHZ	MT	59.67 a	21.17 b	19.16 a	28.55 b	2.78 b
	LNT	59.97 a	21.29 b	18.74 a	29.71 b	2.97 ab
	HNT	60.49 a	22.12 a	17.39 b	35.47 a	3.33 a

Different lowercase letters indicate a significance at *p* < 0.05 for the same cultivars in the same column under varying management. Abbreviations: JXZ, Jiuxiangzhan; HHZ, Huanghuazhan. MT, medium temperature; LNT, low nighttime temperature; HNT, high nighttime temperature.

**Table 3:** Effects of different nighttime temperatures on pasting properties of rice starch.

Cultivars	Treatments	PV (cP)	TV (cP)	BD (cP)	FV (cP)	SB (cP)	Pt (min)	PaT (°C)
JXZ	MT	3347 c	1909 c	1416 a	2764 c	−583 c	6.04 b	79.2 a
	LNT	3401 b	1990 b	1410 a	2860 b	−541 b	6.07 b	78.2 b
	HNT	3453 a	2096 a	1357 a	2997 a	−456 a	6.21 a	77.9 c
HHZ	MT	3829 b	2124 ab	1705 a	3187 b	−641 ab	5.95 a	77.9 b
	LNT	3906 a	2174 a	1732 a	3279 a	−627 b	5.96 a	77.9 b
	HNT	3665 c	2074 b	1591 b	3079 c	−586 a	5.94 a	78.1 a

Different lowercase letters indicate a significance at *p* < 0.05 for the same cultivars in the same column under varying management. Abbreviations: JXZ, Jiuxiangzhan; HHZ, Huanghuazhan. MT, medium temperature; LNT, low nighttime temperature; HNT, high nighttime temperature; PV, peak viscosity; TV, trough viscosity; BD, breakdown viscosity; FV, final viscosity; SB, setback viscosity; Pt, pasting time; PaT, pasting temperature.

**Table 4:** Effects of different nighttime temperatures on thermal properties of rice starch.

Cultivars	Treatments	ΔH(J/g)	T_o_ (°C)	T_p_ (°C)	T_c_ (°C)
JXZ	MT	6.16 a	67.70 a	74.86 b	81.65 a
	LNT	5.97 a	66.70 b	73.27 b	80.20 b
	HNT	6.38 a	68.10 a	75.07 a	81.83 a
HHZ	MT	5.32 a	68.10 a	73.90 b	80.70 b
	LNT	5.56 a	66.43 b	73.20 c	81.40 b
	HNT	5.54 a	68.00 a	75.33 a	82.23 a

Different lowercase letters indicate a significance at *p* < 0.05 for the same cultivars in the same column under varying management. Abbreviations: JXZ, Jiuxiangzhan; HHZ, Huanghuazhan. MT, medium temperature; LNT, low nighttime temperature; HNT, high nighttime temperature; ΔH, enthalpy of gelatinization; T_o_, onset temperature; T_p_, peak temperature; T_c_, conclusion temperature.

**Table 5:** Relationship between chalkiness and the structural and physicochemical properties of rice starch.

Index	Chalky Grain Rate (%)	Chalkiness Degree (%)
Amylose content (%)	−0.971 **	−0.977 **
V < 10 μm	0.839 *	0.838 *
V > 10 μm	−0.839 *	−0.838 *
S < 10 μm	0.922 **	0.923 **
S > 10 μm	−0.922 **	−0.923 **
N < 10 μm	0.842 *	0.841 *
N >10 μm	−0.842 *	−0.841 *
Peak1	0.583	0.572
Peak2	0.773	0.789
Peak3	−0.953 **	−0.960 **
Crystallinity degree (%)	−0.172	−0.157
Branching degree (%)	0.865 *	0.866 *
ΔH(J/g)	0.909 *	0.923 **
T_p_ (°C)	0.215	0.249
T_o_ (°C)	0.018	0.04
T_c_ (°C)	0.025	0.062
PV (cP)	−0.869 *	−0.877 *
TV (cP)	−0.515	−0.524
BD (cP)	−0.957 **	−0.964 **
FV (cP)	−0.731	−0.738
SB (cP)	0.917 *	0.922 **
Pt (min)	0.905 *	0.911 *
PaT (°C)	0.219	0.237

* and ** represent the significance at *p* < 0.05 and *p* < 0.01 levels (2-tailed), respectively. Abbreviations: V, volume percentage; S, surface area percentage; N, number percentage; ΔH, enthalpy of gelatinization; T_o_, onset temperature; T_p_, peak temperature; T_c_, conclusion temperature; PV, peak viscosity; TV, trough viscosity; BD, breakdown viscosity; FV, final viscosity; SB, setback viscosity; Pt, pasting time; PaT, pasting temperature.

## Data Availability

The original contributions presented in the study are included in the article, further inquiries can be directed to the corresponding author.

## Appendix A

**Table A1:** Temperature setting of different nighttime temperature treatments (°C).

Daytime Period	NT	LNT	HNT	Nighttime Period	NT	LNT	HNT
06:00–08:00	27	27	27	18:00–20:00	30	25	35
08:00–10:00	28	28	28	20:00–22:00	29	24	34
10:00–12:00	29	29	29	22:00–24:00	28	23	33
12:00–14:00	32	32	32	00:00–02:00	27	22	32
14:00–16:00	31	31	31	02:00–04:00	26	21	31
16:00–18:00	30	30	30	04:00–06:00	25	20	30

Abbreviations: NT, normal temperature; LNT, low nighttime temperature; HNT, high nighttime temperature.
